# F3MB(PANDER) Decreases Mice Hepatic Triglyceride and Is Associated with Decreased DGAT1 Expression

**DOI:** 10.1371/journal.pone.0117156

**Published:** 2015-02-13

**Authors:** Xiaoqing Mo, Chijiao Yang, Xuelan Wang, Brant R. Burkhardt, Yangbin Li, Haipeng Xia, Xiaopei Cao

**Affiliations:** 1 Department of Endocrinology, First Affiliated Hospital, Sun Yat-sen University, No.58 Zhongshan 2nd Road, Guangzhou 510080, PR.China; 2 Department of Pharmacology, School of Medicine, Sun Yat-sen University, No.74 Zhongshan 2nd Road, Guangzhou 510080, PR.China; 3 Department of Cell Biology, Microbiology and Molecular Biology, University of South Florida, 4202 E. Fowler Avenue, BSF 206, Tampa, FL33620-5550, United States of America; Northeast Ohio Medical University, UNITED STATES

## Abstract

**Objective:**

Pancreatic-derived factor (PANDER, also named as FAM3B) is secreted by pancreatic α and β cells. Increasing evidence suggests that it may serve a hormonal function related to glycemic and lipid metabolism. In this study, we investigated the effects of PANDER overexpression on hepatic and adipose triglyceride metabolism in high-fat diet-fed male C57BL/6 mice.

**Methods:**

PANDER overexpression was achieved by tail-vein injection of recombinant Ad-PANDER and Ad-GFP injected mice served as a control. The TG metabolism in both groups were compared.

**Results:**

Adenoviral-mediated overexpression of PANDER did not affect body weight, food consumption, or liver enzymes. The triglyceride (TG) content of both liver and adipose tissue was significantly decreased in Ad-PANDER mice (liver: 6.16±1.89 mg/g vs. control 14.95±2.27 mg/g, P<0.05; adipose: 39.31±1.99 mg/100mg vs. 47.22±2.21 mg/100mg, *P*<0.05). The free fatty acid (FFA) content of adipose tissue in Ad-PANDER mice was also decreased (1.38±0.18 mg/g vs. 2.77±0.31 mg/g, *P*<0.01). The investigation of key enzymes of triglyceride hydrolysis and FFA oxidation in liver and adipose tissue showed that p-HSL/HSL was significantly increased and that DGAT1 gene and protein expression were significantly reduced in the liver of PANDER-overexpressing mice. PKA phosphorylation was also significantly increased in the livers of Ad-PANDER mice. No differences in ATGL, CPT1, ACOX1, or DGAT2 expression were observed.

**Conclusion:**

Overexpression of PANDER is associated with observable decreases in TG, increases in PKA phosphorylation, and decreased DGAT1 expression, suggesting a possible interrelationship. The mechanisms by which this occurs remain to be elucidated.

## Introduction

Triglycerides (TG) are the most abundant lipids in the mammalian body. Nonesterified fatty acids (NEFA), one of the products of TG hydrolysis, are biomolecules that play multiple roles in energy utilization, receptor signaling and/or intracellular signaling. Inappropriate elevations in NEFA concentrations are toxic and are related to insulin resistance and pancreatic β-cell dysfunction. To avoid toxicity, NEFA are re-esterified into TG for energy storage. The liver is the essential organ for TG synthesis, and white adipose tissue is highly efficient at TG storage. Normal regulation of TG lipolysis and synthesis in liver and white adipose tissue maintains appropriate tissue concentrations of NEFA and whole-body energy homeostasis. Enzymes such as acyl-CoA:diacylglycerol acyltransferase (DGAT) in TG synthesis [1], hormone-sensitive lipase (HSL) and adipose triglyceride lipase (ATGL) in TG mobilization, and carnitine palmitoyltransferase (CPT1) and palmitoyl-CoA oxidase (ACOX1) in fatty acid β-oxidation play essential roles in the regulation of triglyceride metabolism. Their activities are regulated by both hormones and cytokines [2–4].

PANDER, a protein that is primarily expressed by and secreted from α and β cells of the pancreas, belongs to the superfamily of eukaryotic FAM3 proteins. PANDER co-localizes with insulin in insulin-containing secretary granules in β-TC3 and rodent islet β cells [5–7]. Overexpression of PANDER induces apoptosis of pancreatic β cells of mice, rats and humans in a time- and dose-dependent manner via the activation of caspase-3 [6, 8]. Studies in vivo and in vitro have also suggested that PANDER plays a role in the regulation of glucose and lipid metabolism [9–11]. However, the in vivo studies on PANDER overexpression animal models presented controversial results regarding hepatic glycemic and lipid metabolism. In the two reported hepatic PANDER overexpression models by adenoviral delivery, one displayed increased gluconeogenic gene expression and fasting glucose, insulin and corticosterone levels, along with reduced hepatic TG content [11]. In contrast, another study demonstrated increased hepatic TG content and liver lipogenesis in C57BL/6J mice four days after adenoviral tail-vein-delivered hepatic PANDER with no increase of fasting glucose and corticosterone levels, and no glucose intolerance was observed [12]. A recent study on a endocrine pancreas tissue-specific PANDER transgenic mouse has shown elevated fasting glucose, hepatic glucose product and TG content, as well as glucose intolerance[11]. Our previous study has demonstrated that Palmitic acid increases the expression of PANDER in cultured β-TC3 cells in a dose- and time-dependent manner [13]. Although somewhat contradictory, these results suggest that PANDER impacts lipid and glucose metabolism, especially with respect to liver TG homeostasis. The differences in the results obtained in different studies may be attributed to differences in the animal models used, the mode of PANDER delivery, and/or the time of observation.

Hyperlipidemia is a critical detrimental component of both metabolic syndrome and type 2 diabetes mellitus (T2DM). It is mostly accompanied by obesity, fatty liver and T2DM. The observed selective hepatic insulin resistance that occurs in T2DM results in a highly pathological condition of both increased gluconeogenesis and lipogenesis. The triggering molecule, if one exists, for this series of events is unknown. Recent studies on PANDER have presented interesting and controversial data on its effects on both hepatic glucose output and lipid production. For the purpose to further characterize the role of PANDER in hepatic signaling and its potential role in lipid metabolism, especially in the context of obesity, we measured TG metabolism in both liver and adipose tissue of PANDER-overexpressing high-fat diet-fed obese mice.

## Materials and Methods

### Materials and reagents

p-AdEasy-1 vector, p-Shuttle-IRES-hrGFP-1, and BJ5183 electroporation-competent cells were purchased from Stratagene Corporation (La Jolla, CA, USA). Lipofectamine 2000 Reagent (Invitrogen, NY, USA) was used to deliver DNA for transfection. For immunohistochemical assays, anti-FLAG M2 polyclonal antibody (Cell Signaling Technology, 2368) was used. Rabbit PANDER polyclonal antibody (Santa Cruz Biotechnology, sc-83250) was used to detect endogenous and overexpressed PANDER by western blotting. To assess the effects of PANDER on key enzymes of triglyceride lipolysis, the following antibodies were employed: HSL (Santa Cruz Biotechnology, sc-74489), p-HSL (Cell Signaling Technology, 4139), ATGL (Cell Signaling Technology, 2439), DGAT1 (Abcam, ab54037), p-PKA-Thr197 (Cell Signaling Technology, 5661), PKA (Cell Signaling Technology, 5842), and anti-perilipin (Cell Signaling Technology, 9349).

### Construction of Ad-PANDER

The wild-type full-length mouse PANDER gene was cloned into a kanamycin-resistant pShuttle-IRES-hsGFP-1 plasmid containing a cytomegalovirus (CMV) promoter with *Sal*I and *Xho*I restriction enzyme sites using PCR products prepared from restriction endonuclease fragments. Then, 1.0 μg of pShuttle vector plasmid was linearized with *Pme*I and purified by standard cesium chloride/ethidium bromide equilibrium density gradient centrifugation. Purified linearized DNA was then co-transformed with pAdEasy-1 DNA into electrocompetent *E*.*coli* BJ5183 cells. Successful transformants were cultured in LB broth with kanamycin. Following restriction digestion with *Pac*I to confirm the insertion and orientation of the fragment, the plasmid DNA was transformed into DH5α cells for the preparation of large-scale plasmid DNA. Linear DNA was transfected into QBI-293 cells at 50–70% confluency. The recombinant adenovirus vector (Ad-PANDER) was packaged and amplified in HEK-293 cells and purified by CsCl gradient ultracentrifugation. The viral titer was measured by absorbance at 260 nm.

### Animals, diets, and adenoviral administration

This study was carried out in strict accordance with the recommendations in the Guide for the Care and Use of Laboratory Animals of the National Natural Science Foundation. The protocol was approved by the Committee on the Ethics of Animal Experiments of the Sun Yat-sen University (Permit Number: A-004). All surgery was performed under 2% sodium pentobarbital (0.1ml / g mouse) anesthesia, and all efforts were made to minimize suffering. Male 6-week-old C57BL/6J mice (weight 20g to 25g) were fed a high-fat diet (60% of calories from fat, D12492, Research Diets, Inc., New Brunswick, NJ, USA). The mice had access to water *ad libitum*. All mice were housed in an animal facility at Sun Yat-sen University with a 12-h light-dark cycle at 23±1°C. After 6–7 weeks, the mice were divided into two groups and injected with 4.4–5.0×10^10^ vp of Ad-PANDER or Ad-GFP through the tail vein, with additional injections of adenovirus vector five days and ten days later. Surgeries were performed two weeks after the first injection [14]. Food consumption, body weight, and weight gain were measured weekly.

### RNA extraction and RT-PCR

Total RNA was extracted from mouse pancreatic tissue using TRIzol Reagent (Invitrogen, Carlsbad, CA) according to the manufacturer’s protocol. The RNA concentration and purity were determined by using the A260 and A260/A280 ratio in an Agilent Bioanalyzer. cDNA was synthesized from RNA with random hexamer primers and Moloney murine leukemia virus (M-MuLV) reverse transcriptase (Fermentas, St. Leon-ROT, Germany) following the manufacturer’s instructions. The resulting cDNA was amplified by PCR using the primer sequences below (*Sal*I and *Xho*I restriction sites are underlined):

PANDER-F: AATTGTCGACGCCACCATGCGTCCAGTTGCTACAGGC and

PANDER-R: AATTCTCGAGTCTCAGCCCTTTGGGTATGCA.

The PCR amplification process consisted of five minutes of denaturation at 94°C followed by 35 cycles of 45 sec of denaturation at 94°C, 45 sec of annealing at 60°C, and 45 sec of extension at 72°C. The PCR products were separated and visualized by agarose gel electrophoresis.

### Real-time RT-PCR

Total RNA was isolated from mouse liver and adipose tissue using TRIzol Reagent and reverse-transcribed using the ReverTra Ace qPCR RT Kit for real-time PCR (TOYOBO, JAPAN) according to the manufacturer’s instructions. The PCR mix included THUNDERBIRD SYBR qPCR Mix (10 μl), forward primers and reverse primers (0.6 μl each), 50× ROX reference dye (0.4 μl), DNA (2 μl), and PCR-grade water to a final volume of 20 μl. The primers used were as follows: mouse

*Dgat1*: forward, 5’- TTTGCTCTGGCATCATACTCC-3’, reverse, 5’- CCACTGACCTTCTTCCCTGTA-3’; mouse DGAT2: forward, 5’- ACGCAGTCACCCTGAAGAAC-3’, reverse, 5’- CCAAAGGAATAAGTGGGAACC-3’; mouse

*Cpt1*: forward, 5’- CTCCGCCTGAGCCATGAAG-3’, reverse, 5’- CACCAGTGATGATGCCATTCT-3’; mouse ACOX1: forward, 5’- CCGCCACCTTCAATCCAGAG-3’, reverse, 5’- CAAGTTCTCGATTTCTCGACGG-3’; and mouse β-actin:

forward, 5’-GGCTGTATTCCCCTCCATCG-3’, reverse, 5’- CCAGTTGGTAACAATGCCATGT-3’. The real-time PCR cycling conditions were as follows: initial denaturation at 95°C for 60 sec followed by 40 cycles of denaturation at 95°C for 15 sec and elongation at 60°C for 45 sec (Real-Time PCR 7500, ABI, USA). Target gene mRNA expression levels were normalized to β-actin, and expression levels relative to control were measured using the 2^-ΔΔCt^ method [15].

### Western blotting assay

One milliliter of lysis buffer (ProteoJET Mammalian Cell Lysis Reagent, Fermentas, K0301) containing 10 μl protease inhibitor cocktail (Thermo, 78440) was added to a 100-mg piece of hepatic or adipose tissue, which was then disrupted with an electric homogenizer at 4°C. After centrifugation for 20 min at 12,000 rpm at 4°C, the supernatant was collected and placed in a fresh tube. Protein concentration was determined by the Bradford method. Samples were frozen at -20°C for later use. The levels of PANDER, ATGL, HSL, p-HSL, DGAT1, PKA, p-PKA-Thr197, and PERILIPIN were measured by western blotting after electrophoresis on a 7 or 12% sodium dodecyl sulfate-polyacrylamide gel. The electrophoresed proteins were electrotransferred to polyvinylidene difluoride membranes. The membranes were incubated with primary antibodies for 12–16 hours at 4°C and with HRP-conjugated secondary antibodies at room temperature with agitation for 1 hour. Proteins were detected by chemiluminescence with ECL (Invitrogen), with quantitation by densitometry.

### Immunohistochemistry

Pancreas, liver, heart, lung, kidney, spleen, testis, and small intestine isolated from male C57BL/6 mice that had been transfected with the Ad-PANDER vector were removed and immediately fixed in formalin. The tissue was then dehydrated and embedded in paraffin, and 4-μm sections were mounted on ProbeOn Plus slides (FisherBiotech, 15-188-52). The slides were stored at room temperature until staining. After eliminating the endogenous peroxidase with 0.3% H_2_O_2_ in methanol, sections were incubated with rabbit anti-Flag for 30 min at room temperature and then incubated for 30 min with EnVision+, Peroxidase, Rabbit (Santa Cruz, USA). The sections were developed with 3,3-diaminobenzidine (DAB) without light for 10 min, followed by washing in distilled water. The slides were then counterstained with hematoxylin and immediately dehydrated, cleared, and mounted with neutral gum. The slides were visualized using a confocal microscope (Leica, DM IRE2) coupled to a spectral confocal system (Leica, TCS SP2), and photomicrographs were taken.

### Folch method for total lipid determination in liver and adipose tissue

Total lipids were extracted from liver and adipose tissue of mice using the Folch method [16]. In brief, 100 mg of tissue was homogenized in 2 ml chloroform/methanol (2/1); the mixture was then agitated in an orbital shaker at room temperature for 20 min and centrifuged. The lower phase, which contained most of the tissue lipids, was subsequently recovered, and the solvent was washed with 400 μl of 0.9% NaCl solution. The mixture was centrifuged at low speed to separate the two phases. The lower, chloroform phase containing the lipids was recovered and evaporated under vacuum. The recovered lipids were dissolved in 1 ml isopropanol, and the TG and FFA contents was determined with enzymatic (GPO-POD) and colorimetric (ACS·ACOD) methods, respectively, using a Hitachi automatic biochemical analyzer.

### Oil red O staining

Frozen liver tissue sections 10 μm thick were cut and mounted on slides. After air-drying at room temperature, the slides were fixed in ice-cold formalin for 10 min and rinsed immediately in distilled water three times. After air-drying again, the slides were stained for 10–15 min with freshly prepared Oil Red O working solution and rinsed with 60% isopropanol. After staining in Mayer’s hematoxylin for 30–60 sec, the slides were washed thoroughly in distilled water, mounted with glycerin jelly, and photographed under an electron microscope (Olympus, Japan).

### Measurement of serum metabolites

To evaluate serum metabolites, blood from the sacrificed animals was collected in an Eppendorf tube, incubated at room temperature for 30 min, and centrifuged at 4,000 rpm for 15 min at 4°C. The serum was removed and stored at -80°C until further analysis. Insulin and PANDER levels were determined using commercially available ELISA kits (Millipore, Uscn). Total serum cholesterol concentration was determined with a cholesterol assay kit (Nanjing JianCheng). Antigen-antibody complex direct assays (Nanjing JianCheng) were used to measure serum LDL-C and HDL-C. Serum, liver, and adipose tissue levels of TG and FFA were determined by enzymatic (GPO-POD) and colorimetric methods (ACS·ACOD), respectively.

### Statistical analysis

All data were analyzed using SPSS 16.0 (Chicago, IL USA). Data are presented as the mean ± SEM. Significant differences between groups were analyzed by Student’s *t* test, and a two-tailed *P*<0.05 was considered statistically significant.

## Results

### Construction and confirmation of adenoviral PANDER

To study the effect of PANDER *in vivo*, an adenovirus vector containing the full-length murine PANDER gene (Ad-PANDER) was constructed using the Ad-Easy XL system and confirmed by DNA sequencing. Ad-PANDER and Ad-GFP were packaged and amplified in 293 cells. One week after transfection, The 293 cells were stained with bright green which refers to the expression of EGFP as observed under the fluorescence microscope as well as the phase contrast microscope. The total protein of 293 cells with amplified Ad-PANDER were collected and PANDER expression were measured by western blot. As expected Ad-PANDER strongly expressed PANDER (26KD) and PANDER with Flag (29KD).

### Hepatic PANDER overexpression in mice

After 6–7 weeks of high-fat diet feeding (HFD) (60% of calories from fat), obese mice with glucose intolerance and insulin resistance according to the results of Intraperitoneal glucose tolerance test (IPGTT) and Intraperitoneal insulin tolerance test (IPITT) before and after HFD were obtained.

**Fig 1:**
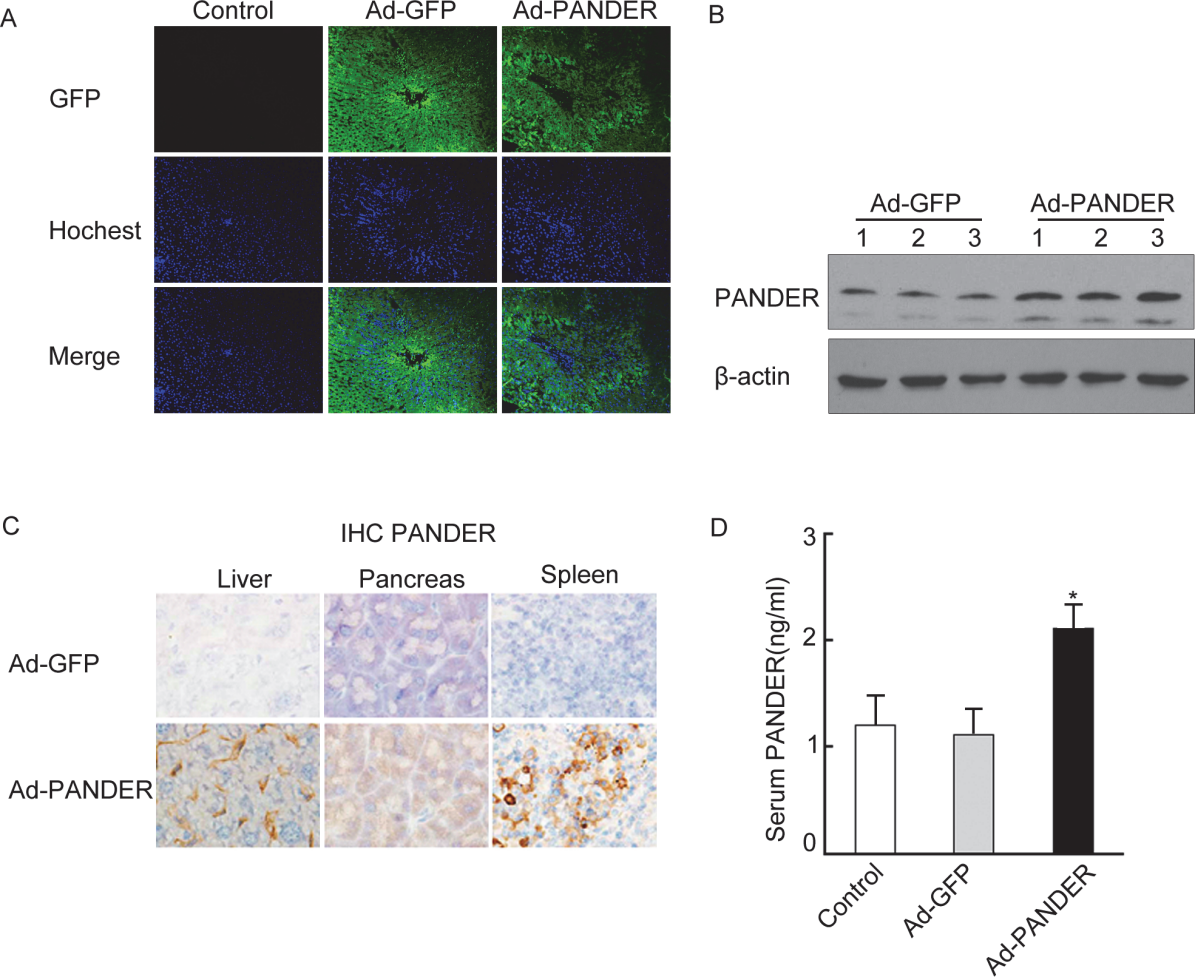
Expression of specific proteins in PANDER-overexpressing C57BL/6 mice. C57BL/6 mice were injected with 4.4–5.0×10^10^ vp of Ad-PANDER or Ad-GFP via the tail vein after 6–7 weeks on a high-fat diet. Tissue samples were collected 14 days after adenovirus injection. (A) GFP expression in the liver of mice after adenovirus injection (100×). (B) Western blot analysis demonstrating increased PANDER in liver of PANDER-overexpressing mice. *P<0.05 vs. Ad-GFP, *n* = 5. (C) Immunohistochemical detection of Flag in liver of mice after adenovirus transfection (200×). (D) Serum PANDER was increased in Ad-PANDER-infected mice. *P<0.05 vs. Ad-GFP, *n* = 5.

To determine the effect of PANDER on TG metabolism, we used Ad-PANDER to induce overexpression of PANDER in male C57BL/6 HFD mice via tail vein injection. Adenoviral transgene delivery via tail vein specifically targets the liver (Herrmann *et al*., 2004). Therefore, we initially confirmed hepatic-specific PANDER overexpression. Mice transfected with either Ad-PANDER or the Ad-GFP adenovirus showed specific expression of GFP, Flag, and PANDER in the liver and serum. GFP was expressed in both Ad-PANDER and Ad-GFP mice (Fig. 1A). Hepatic PANDER protein was increased 3.7-fold in Ad-PANDER-injected mice compared with Ad-GFP-injected mice (Fig. 1B). Flag was recognized in the liver of Ad-PANDER mice (Fig. 1C). In addition, serum PANDER was increased approximately two-fold in Ad-PANDER mice compared with Ad-GFP mice (Fig. 1D).

### Ad-PANDER mice exhibit similar body weight, weight gain, food consumption, and liver enzymes

**Fig 2:**
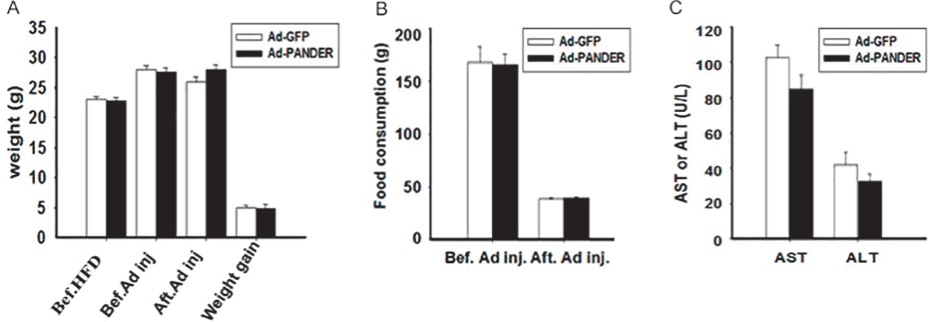
Body weight, weight gain, food consumption, and liver enzymes in high-fat diet PANDER-overexpressing mice. (A) Average body weight and weight gain. (B) Food consumption per mouse in the Ad-PANDER and Ad-GFP groups. (C) Liver enzymes AST and ALT in Ad-PANDER and Ad-GFP mice. Values are means ± SEM (*n* = 5). Bef HFD: before high-fat diet; Bef Ad inj: before adenovirus injection; Aft Ad inj: two weeks after adenovirus injection; ALT: alanine aminotransferase; AST: aspartate aminotransferase.

Adenoviral-mediated overexpression of PANDER did not affect body weight, food consumption, or liver enzymes, as determined by weekly measurements and biochemical analysis (Fig. 2A to C).

### Serum lipid profile in high-fat diet-fed Ad-PANDER mice

As shown in Table 1, Ad-PANDER mice fed a high-fat diet had lower serum FFA than Ad-GFP mice (395.7±16.35 vs. 552.2±42.82, *P*<0.05). The TG level tended to be lower in the Ad-PANDER mice than the controls although there was no statistical significance. No differences in serum CHO, HDL, or LDL were observed. The Ad-PANDER mice had higher fasting glucose level and significantly increased fasting insulin level than that in the Ad-GFP mice

**Table 1:** Effects of PANDER on fasting serum biochemical parameters of mice.

**Serum chemistry**	**Ad-GFP (n = 5)**	**Ad-PANDER (n = 5)**	***P*-value**
Glucose (mmol/L)	9.2±0.97	12.9±2.22	0.069
CHO (mmol/L)	2.60±0.15	2.84±0.17	0.231
TG (mmol/L)	0.516±0.45	0.39±0.60	0.238
HDL (mmol/L)	1.47±0.47	1.62±0.12	0.188
LDL (mmol/L)	0.144±0.29	0.174±0.19	0.334
FFA (μmol/L)	552.2±42.82	395.7±16.35	0.048
Insulin(nmol/L)	311.98±16.85	388.02±20.37	0.01

### Decreased TG content in the liver and adipose tissue of Ad-PANDER mice

**Fig 3:**
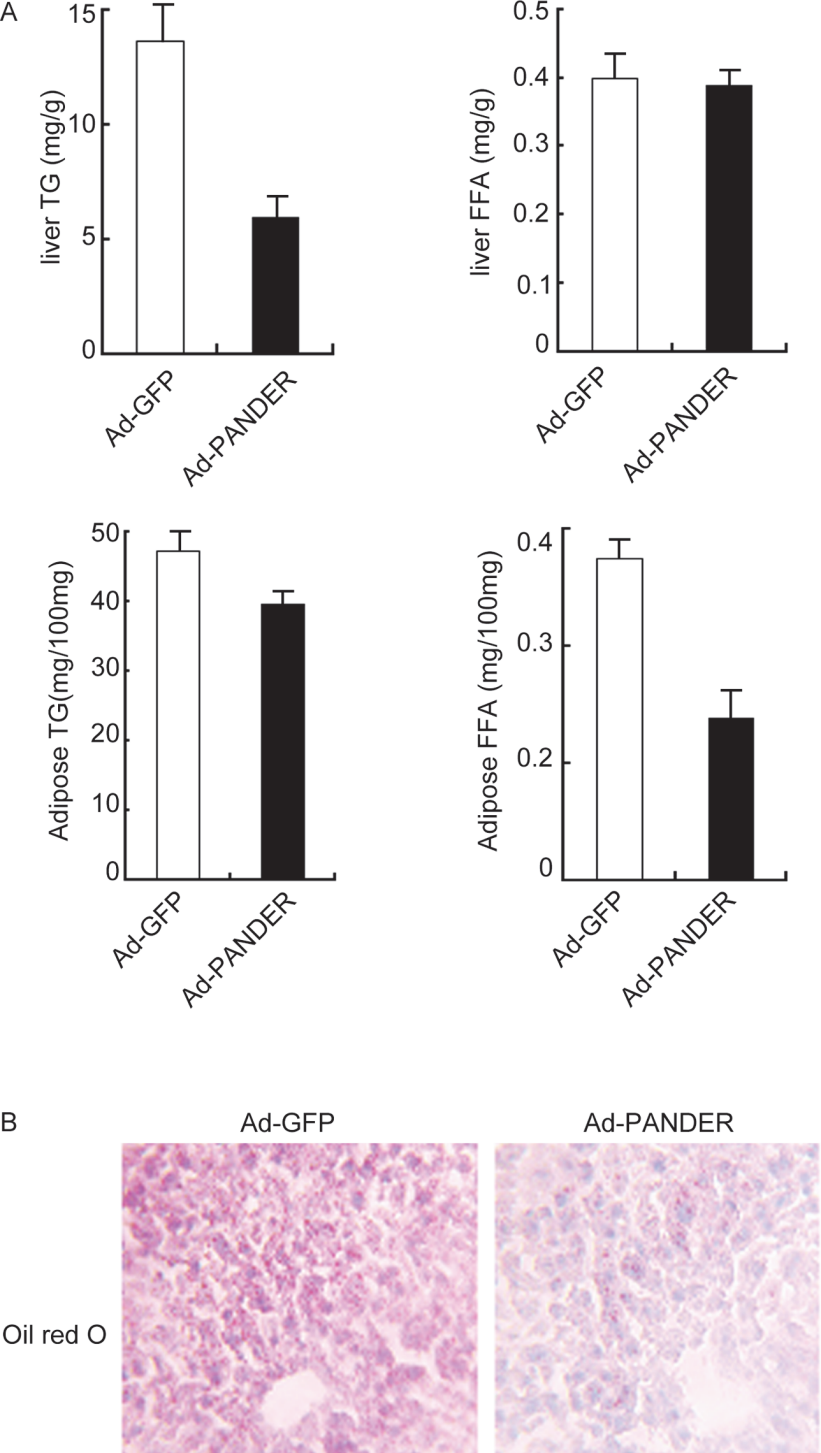
Effects of PANDER overexpression on the TG and FFA contents of the liver and adipose tissue of mice. (A) TG and FFA were measured as described in the Methods. The results are represented as the mean ± SEM of five independent samples. * *P*<0.05 vs. Ad-GFP. # P<0.01 vs. Ad-GFP. *n* = 5. (B) Liver TG content was measured with Oil red O staining.

The lipid contents of liver and adipose tissue were determined 14 days after adenovirus infection. Both TG contents were significantly decreased in Ad-PANDER mice compared with control animals (liver: 6.16±1.89 mg/g vs. 14.95±2.27 mg/g, *P*<0.05; adipose: 39.31±1.99 mg/100mg vs. 47.22±2.21 mg/100mg, *P*<0.05); the FFA content of adipose tissue was also significantly decreased (1.38±0.18 mg/g vs. 2.78±0.31 mg/g, *P*<0.01). No difference in FFA level in the livers of Ad-PANDER and Ad-GFP-injected mice was observed (Fig. 3A). Oil Red O staining also indicated significantly decreased liver TG content in the high fat-fed Ad-PANDER mice (Fig. 3B).

### Effects of PANDER on the key enzymes of triglyceride lipolysis

**Fig 4:**
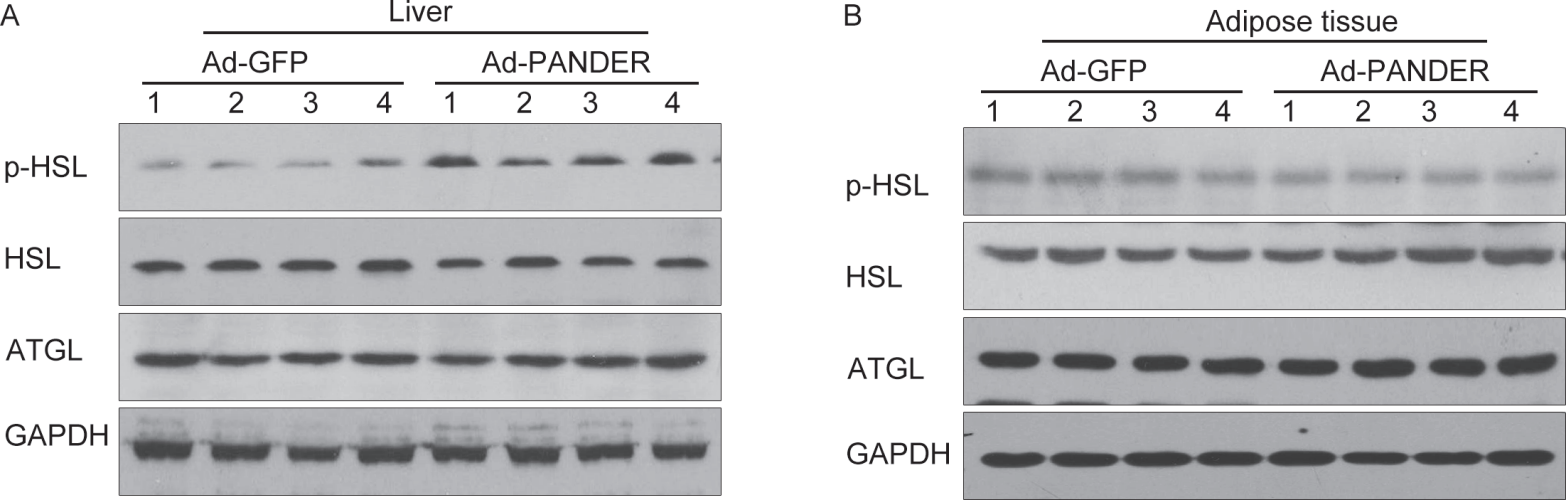
Effects of PANDER overexpression on key enzymes of triglyceride hydrolysis in liver and adipose tissue of mice. Total HSL, p-HSL, and ATGL were measured in liver (Fig. 4A) and adipose tissue (Fig. 4B) by western blot 14 days after adenovirus transfection. The results are presented of five independent samples.

To explore the possible mechanism underlying the effects of PANDER on TG metabolism, we measured the total and phosphorylated levels of key enzymes of triglyceride hydrolysis in liver and adipose tissue. Among triglyceride lipases, HSL and ATGL are the two essential lipases [17]. We measured the expression of ATGL, HSL, and p-HSL by western blot analysis. The results showed that p-HSL was significantly increased (*p*<0.05) in liver of Ad-PANDER mice. No difference in ATGL and total HSL expression in liver was observed between the Ad-PANDER mice and Ad-GFP mice (Fig. 4A). The expression of p-HSL, HSL, and ATGL in adipose tissue also did not differ significantly between the two groups (Fig. 4B).

### PANDER increases liver PKA phosphorylation in high-fat diet mice

**Fig 5:**
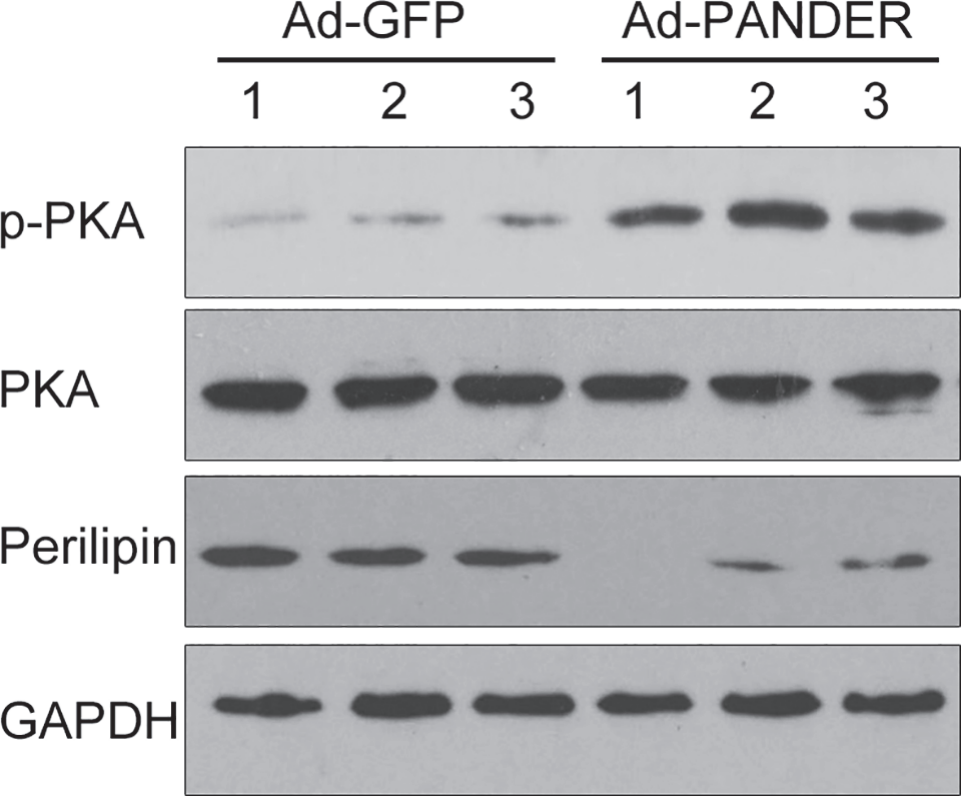
Effect of PANDER overexpression on the PKA pathway in the mouse liver. Total PKA, p-PKA, and PERILIPIN in the liver of Ad-GFP- and Ad-PANDER-infected high-fat diet-fed mice were measured by western blot 14 days after adenovirus transfection.The results are presented of five independent samples. * *P*<0.05 vs. Ad-GFP.

To further explore the effect of PANDER on TG metabolism, we evaluated the effects of PANDER on PKA signaling in the liver of HFD mice. Western blot analysis demonstrated that hepatic p-PKA was significantly increased by PANDER overexpression. Furthermore, the expression of the downstream protein perilipin was significantly decreased in Ad-PANDER mice compared with Ad-GFP mice (Fig. 5).

### PANDER suppresses the expression of hepatic DGAT1 in high-fat diet mice

**Fig 6:**
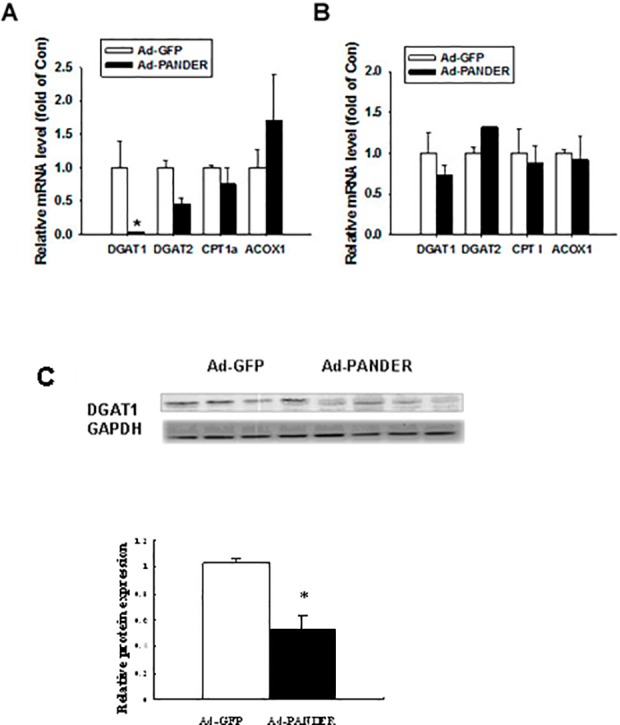
Effects of PANDER on the mRNA expression of *Dgat1*, *Dgat2*, *Cpt1*, and *Acox1* in the liver and adipose tissue of mice. (A) Real-time PCR analysis of *Dgat1*, *Dgat2*, *Cpt1*, and *Acox1* in the liver. (B) Real-time PCR analysis of *Dgat1*, *Dgat2*, *Cpt1*, and *Acox1* in adipose tissue. The results are represented as the mean ± SEM of five independent samples. (C) Western blot analysis of DGAT1 in liver. *P<0.05 vs. Ad-GFP.

DGAT, which includes the two enzymes, DGAT1 and DGAT2, is the key enzyme in the regulation of triglyceride synthesis (Farese *et al*.,2000), while ACOX1 and CPT1 are the key enzymes of FFA β-oxidation. To assess the effects of PANDER on triglyceride synthesis and FFA oxidation, the mRNAs for *Dgat1*, *Dgat2*, *Acox1* and *Cpt1* were measured using real-time PCR analysis. No differences in the mRNA level of *Dgat2*, *Acox1*, or *Cpt1* in liver or adipose tissue between Ad-PANDER and Ad-GFP mice were observed (Fig. 6A, 6B). However, both the mRNA (0.32±0.03 vs Ad-GFP 1.00±0.39, P<0.05) and protein expression (0.53±0.18 vs Ad-GFP 1.03±0.07, P<0.05) of hepatic DGAT1 were significantly downregulated by PANDER overexpression (Fig. 6A, 6C), although no difference in DGAT1 expression in adipose tissue was observed (Fig. 6B).

## Discussion

Obesity is one of the most prevalent diseases in modern society; it affects approximately one-tenth of the population in the United States [18, 19]. Obesity is typically accompanied by hyperlipidemia, hyperglycemia and hyperinsulinemia, along with many other organ dysfunctions, and can cause tremendous physiological and psychological problems, including the onset of type 2 diabetes [20]. Hormones and cytokines participate directly or indirectly in the regulation of the development of metabolic disorder [21]. The pancreatically derived cytokine PANDER plays a role in the regulation of glucose metabolism and promotion of insulin resistance [6, 10, 11, 22]. PANDER may also regulate lipid metabolism *in vitro* and *in vivo*[11–13]. However, conflicting results have been reported about PANDER. To gain a better understanding of the effects of PANDER on lipid metabolism, especially in the context of obesity, we evaluated the effect of PANDER on TG metabolism in high-fat diet-fed obese mice. Our data indicate that PANDER may have a suppressive effect on lipogenesis in both liver and adipose tissue. In our model, hepatic overexpression of PANDER did not alter body weight, weight gain, or liver function.

To date, evidence regarding the impact of PANDER on hepatic insulin signaling and triglyceride synthesis is sparse and presents a conflicting story. Two prior studies have explored how Ad-PANDER delivery alters insulin signaling, glycemic regulation, and triglyceride synthesis. Using adenovirus delivery, Li *et al*. (2011) demonstrated a 100-fold upregulation of hepatic PANDER mRNA and four-fold upregulation of hepatic PANDER protein as compared to controls. No data were presented confirming the overexpression of PANDER in the serum. Phenotypic analysis in that study was performed 3 days after viral delivery. Their Ad-PANDER mice demonstrated hepatic steatosis with increased hepatic and serum triglycerides. Although no difference in glucose tolerance was noted, suppressed Akt signaling and increased gluconeogenic expression were observed. In contrast, Wilson *et al*.(2010) demonstrated 1.5-fold upregulation of hepatic PANDER in Ad-PANDER mice, with 2.7-fold elevation of PANDER in the serum. In that study, phenotypic analysis was performed seven days after viral delivery. No differences in fasting triglycerides were observed. However, hepatic TG decreased in the fasted state. Robert-Cooperman et al. (2014) generated a transgenic mouse that exclusively overexpressing PANDER from the endocrine pancreas. In this animal model, 4 fold increased PANDER serum level was achieved and no changes of hepatic PANDER expression was observed. Fasting glucose and insulin levels were elevated in the young 3 month old mice but not in the 6 month old mice. Phenotype presented increased hepatic insulin resistance with glucose intolerance as well as increased hepatic TG content and with no changes of peripheral insulin sensitivity. These studies differ in several ways, including the differences of animal model, the extent of upregulation of hepatic PANDER, level of PANDER in the serum, and number of days after model generation at which the phenotype was observed.

The apparent conflicting effects of PANDER on lipogenesis were the focus of our study. Our study design included evaluation of the phenotype produced by Ad-PANDER two weeks after viral delivery in high-fat-diet obese mice. Similar to Wilson *et al*.(2010), we observed that PANDER decreased lipid deposition. PANDER is expressed in both liver and white adipose tissue and shows increased expression in the obese state [12]. Therefore, we measured TG metabolism in both liver and adipose tissue. Although the lipid profile of PANDER-overexpressing mice showed slightly decreased serum fasting TG, the difference did not reach statistical significance. No differences in cholesterol, LDL, or HDL were observed. However, serum FFA and the TG content of both liver and adipose tissue were significantly decreased in PANDER-overexpressing mice compared with control animals, and the FFA content of adipose tissue was also significantly decreased.

To further explore the possible mechanism by which PANDER suppresses TG metabolism, key enzymes in TG metabolism were examined. HSL and ATGL are the key enzymes in TG lipolysis; they are highly expressed in adipose tissue and expressed to a lesser extent in the liver. Increased activation of HSL and ATGL promotes FFA β-oxidation and decreases TG deposition in the liver. Our results show that the p-HSL/HSL ratio was significantly increased in the liver of Ad-PANDER mice compared with Ad-GFP mice, suggesting Ad-PANDER increased HSL phosphorylation in the liver. There were no significant differences in HSL or p-HSL in adipose tissue between the Ad-PANDER mice and controls. In addition, the expression of ATGL in liver and adipose tissue was similar in both groups. DGATs are the key enzymes in triglyceride synthesis, and CPT1 and ACOX1 are the key enzymes in FFA β-oxidation. As determined by RT-PCR, PANDER specifically inhibited the gene and protein expression of DGAT1 in the liver of Ad-PANDER mice and had no impact on DGAT2, CPT1, or ACOX1 in liver. DGAT1, DGAT2, CPT1, and ACOX1 showed no differences in adipose tissue between Ad-PANDER mice and Ad-GFP mice. Increased activation of HSL and decreased DGAT1 promotes FFA β-oxidation and decreases TG synthesis in the liver.

Increased activity of HSL in liver enhances TG hydrolysis, resulting in reduced TG content. The cAMP-dependent protein kinase (PKA) pathway plays an important role in the regulation of TG homoeostasis through its effects on HSL phosphorylation and DGAT expression. cAMP activates PKA, which regulates lipolysis by phosphorylating perilipin and HSL and inhibits TG synthesis [23, 24]. Perilipin is localized at the periphery of lipid droplets and serves as a protective coating against lipases [25]. Phosphorylation of perilipin results in a conformational change in the protein that exposes lipid droplets to endogenous lipases, such as HSL [5]. Thus, phosphorylation of HSL and perilipin plays a pivotal role in lipid storage. The increased PKA and HSL phosphorylation as well as decreased total perilipin protein in the Ad-PANDER mice in our study indicate that PANDER may affect TG metabolism through the PKA pathway.

The liver is the primary organ of TG synthesis, and adipose tissue serves as the main area of TG storage. Adipose tissue also functions as an energy reserve for TG lipolysis and FFA oxidation. Because we did not observe any effects of PANDER on adipose tissue, including expression or activation of enzymes involved in TG hydrolysis and TG synthesis, we presume that the effects of PANDER overexpression on the TG and FFA content of serum and adipose tissue are the result of its effects on TG metabolism in the liver, presumably via PANDER-induced inhibition of DGAT1 expression and increased HSL phosphorylation.

Reports indicate that PANDER inhibits Akt phosphorylation [8, 11]. In our model, overexpression of PANDER in high-fat diet-fed obese and pre-diabetic mice resulted in increased serum fasting insulin and a trend increased fasting glucose level. Hepatic overexpression of PANDER did not alter body weight, weight gain, or liver function.

Taken together, our data indicate that PANDER plays a role not only in the regulation of glucose metabolism but also in the regulation of triglyceride synthesis and degradation. Adenovirus-mediated overexpression of PANDER in high-fat diet-fed mice decreased the expression of DGAT1, the key enzyme in TG synthesis, and upregulated p-HSL/HSL, the key enzyme in TG hydrolysis in the liver. Because the observed effects were found specifically in the liver, our results show that PANDER does not impact adipose tissue directly. Our study provides novel insight into the effects and mechanism of action of PANDER in hepatic TG metabolism and provides further evidence regarding the pleiotropic role of this novel secreted cytokine.
